# Perspectives of Teachers on Digital Literacy Implementation Curriculum in Elementary Schools

**DOI:** 10.12688/f1000research.172273.1

**Published:** 2025-11-24

**Authors:** Rusi Rusmiati Aliyyah, Teguh Prasetyo, Widyasari Widyasari

**Affiliations:** 1 Faculty of Teacher Training and Education, Universitas Djuanda, Bogor, West Java, 16720, Indonesia; 2 Faculty of Educational Technology, Universitas Ibn Khaldun, Bogor, West Java, Indonesia

**Keywords:** Curriculum Management, Digital Literacy, Elementary School, Thematic analysis, 21st century skills

## Abstract

The application of digital literacy in elementary schools is a curriculum-driven approach to create independent and student-centered learning through the effective and efficient use of digital technology. This study explores teachers’ perceptions of the application of digital literacy in the curriculum to improve the quality of elementary schools. The research uses a quasi-qualitative approach. Data was collected through structured interviews with 48 teachers from 26 elementary schools, while data analysis used thematic analysis. The results of the study summarize the statements of elementary school teachers that revealed three main themes: the urgency, strategy, and positive and negative impacts of implementing digital literacy in the curriculum in elementary schools. The teachers stated that implementing digital literacy is critical because it impacts the efficiency and effectiveness of the use of school resources, improves the quality of schools, and helps develop 21st-century skills. This study concludes that school principals need to manage the curriculum and prepare regulations that regulate the availability of infrastructure for implementing digital literacy in elementary schools. This study also recommends that the Indonesian government make policies on the rules for implementing digital literacy in elementary schools and elementary schools can implement digital literacy in a sustainable manner and be prepared based on the curriculum according to the potential and culture of the school.

## Introduction

Digital literacy is the knowledge and skills of consumers in utilizing digital media ( Lazonder et al., 2020) to improve skills in the school environment to support cognitive, social, and emotional abilities ( Churchill, 2020; Jang et al., 2021; UNESCO Institute for Statistics, 2018; Van Laar et al., 2017). Research states that digital literacy learning has an impact on 21st-century skills development ( Churchill, 2020; Kim et al., 2014; List, 2019; Purnama et al., 2021; Siddiq et al., 2017) that focuses on competencies so that students have critical thinking skills and good metacognition ( Siddiq et al., 2017).

Various countries have implemented digital literacy learning, which is packaged in the development of a curriculum in accordance with the potential of schools to improve the quality of education in their countries. Selected European countries (Slovakia, Czechia, Poland, the United Kingdom, and Ireland), as well as the United States and Sweden, include digital literacy in primary school students’ learning to improve students’ numeracy literacy skills ( Javorský & Horváth, 2014; UNESCO Institute for Statistics, 2018). The results of the study stated that European countries use six national educational technology standards in basic education, namely creativity and innovation, communication and collaboration, research and information fluency, critical thinking, problem solving, decision making, technology operations and concepts ( Javorský & Horváth, 2014). China has developed a digital learning curriculum to improve the literacy skills of grade 4 elementary school students ( Harvey & Brooks, 2022). Hungary develops a curriculum to improve information literacy skills and competencies ( Varga & Egervári, 2014). Canada designs classroom-based cybersecurity, privacy, and digital literacy games for elementary school students ( Maqsood & Chiasson, 2021). The United States is developing a digital model to make it easier for elementary school teachers to implement technology-based curriculum ( Obel-Omia, 2016). Taiwan develops inquiry and six-frame learning to integrate information literacy into the primary school curriculum ( Chen, 2018). Meanwhile, Korea developed a national curriculum that teaches software learning in the private activities of 5-year-olds ( Lee et al., 2018).

Meanwhile, to improve the quality of elementary schools, especially in digital literacy skills, Indonesia has formed the school literacy movement as a comprehensive effort that involves school residents (teachers, students, and parents of students) and the community to participate in the education ecosystem. Meanwhile, to answer the demands of technology-based 21st century learning, in 2021, the Ministry of Education, Culture, Research, and Technology of the Republic of Indonesia launched a digital literacy module in elementary schools that contains strategies for implementing digital literacy carried out inside and outside the classroom in the form of extracurricular activities with a learning focus on time management, cyberbullying, cybersecurity management, Privacy Management, Critical Thinking, and Digital Empathy ( Rahmah, 2015). Indonesia has also developed a digital corner for technology-based learning activities in all educational units, including elementary schools, it has also developed computational thinking in the curriculum of local school content ( Aliyyah et al., 2024). All of these digital literacy-based learning activities are contained in the curriculum developed by the Indonesian government under the name of the merdeka curriculum. Merdeka curriculum is a curriculum with diverse intracurricular learning with more optimal content so that students have enough time to explore concepts and strengthen competencies. Merdeka curriculum encourages the mastery of digital skills, where students learn to use technology wisely, safely, and critically with the information they encounter ( Emawati et al., 2024).

However, elementary schools in Indonesia have found it very difficult to implement the program the government has launched because of its many problems. Among them are the limited software owned by the school, the low awareness of users in utilizing the facilities owned by them ( Ministry of Education, Culture, 2021), low public awareness and knowledge about Information and Communication Technologies (ICT), there are no special subjects in the elementary school curriculum about ICT so that teachers and students are not given knowledge and understanding about the ethics of using ICT. In addition, there are not enough adequate technological facilities in schools or parenting activities with parents about digital literacy learning ( Rahmah, 2015). Thus, this research is emphasized to answer and provide alternative solutions to the problem.

This study aims to explore teachers’ opinions on the application of digital literacy in the elementary school curriculum in Indonesia. The main questions are:

1. How urgent is the digital literacy implementation curriculum in elementary schools?
2. How teacher’s strategy in digital literacy implementation curriculum in elementary schools?
3. How the positive and negative impact on teachers from the digital literacy implementation curriculum in elementary schools?

## Merdeka curriculum in Indonesia

Merdeka curriculum is a curriculum that has been implemented in Indonesia since February 2022 with a diverse and optimal intracurricular learning design so that students have enough time to explore concepts and strengthen competencies ( Ministry of Education and Culture of the Republic of Indonesia, 2022). Merdeka curriculum provides flexibility for educational units and teachers in designing learning that is in accordance with the learning context and needs and student-centered ( Aliyyah et al., 2023). In addition, merdeka curriculum emphasizes the importance of utilizing various sources of knowledge, not only textbooks but also information obtained through digital platforms and other related references ( Emawati et al., 2024).

## Methodology

### Research design

The approach used in this research was quasi-qualitative with a simple research design. Quasi-qualitative research is a study with the primary objective of objectively describing a situation according to the problem ( Cropley, 2019). Meanwhile, according to Bungin ( Bungin, 2020), quasi-qualitative is a part of research influenced by positivism, which is used in the presentation of theory, a kind of deductive approach, so this research cannot be entirely qualitative. This can be seen during the analysis of the data. So it is included in the quasi-qualitative research. Quasi-qualitative research is suitable for narrating the life of information sources that can be expressed descriptively. One type of quasi-qualitative research is a simple research design (SRD). SRD is a research design used by a researcher to reflect on findings in the field by using theory to solve the problems encountered ( Bungin, 2020). The research procedure of SRD was carried out with five main steps, namely (1) Selecting the social context and determining the research question (Social context and research question); (2) Conducting a literature review (Literature Review); (3) Conducting research methods and collecting data (Research methods and data collection); (4) Analyzing data (Data Analysis); (5) Reporting research results (Reporting) ( Bungin, 2020).

### Participants

The participants in this study were 48 teachers spread across 26 elementary schools in seven provinces in Indonesia, covering the regions of West Java, Jakarta, Central Java, Yogyakarta, East Java, North Sumatra, and South Kalimantan. This is in accordance with Creswell’s opinion about the concept of qualitative research ( Creswell, J. W., & Poth, 2018). The selection of elementary school teachers is based on experience implementing digital literacy in elementary schools for at least three years. Teacher data was taken from seven provinces, based on data from the Ministry of Education and Culture of the Republic of Indonesia, which stated that these seven regions have the best digital literacy users in Indonesia in 2022 and 2023. The purposive sampling technique is used by conducting direct interviews with teachers who have used digital-based learning media in the form of the internet, PowerPoint, YouTube, e-books, learning videos, learning applications, learning games, Google Classroom, Moodle, online learning multimedia, and others that support digital-based learning both inside and outside the classroom at least four times a week. Data collection was carried out from August to October 2024. Descriptive data on demographic characteristics, including gender, age, length of teaching, and level of education, are presented in Table 1.

**Table 1.** Participant’s profiles.

Respondent profile	Frequency	Served (%)
Gender		
Woman	32	67%
Man	16	33%
Age		
20-29	9	19%
30-39	20	42%
40-49	9	19%
50-59	10	20%
Education level		
Bachelor	43	90%
Magister	5	10%
Doctor	0	0%
Long teaching time		
1-5 years	13	27%
6-10 years	3	6%
11-15 years	8	17%
16-20 years	10	21%
21-25 years	8	17%
Over 25 years old	6	12%

In the first stage, the researcher involved principals in elementary schools implementing digital literacy in 7 pre-selected provinces. The principal provides a Google Form link containing questions to the teacher as a respondent, with the criteria for filling out the Google Form. After the data was entered and the initial coding was carried out, the researchers selected five people from 48 respondents as in-depth interview participants to sharpen and confirm the answers not found in the Google Form. The researcher selected the interview participants by checking the answers on the Google Form with the most detailed answer criteria related to the questions and research objectives.

### Instruments

The instrument used in this study is in the form of open-ended questions which are then made in the form of transcripts containing teachers’ ideas or opinions about the application of digital literacy in the curriculum in elementary schools based on their experience so far. The following are the questions given to teachers:

1. Explain the urgency of digital literacy implementation curriculum in elementary schools!
2. Explain teachers’ strategies in digital literacy implementation curriculum in elementary schools!
3. Explain the positive and negative impacts of digital literacy implementation curriculum in elementary schools!

### Data analysis

The data analysis used was a thematic analysis technique to identify, evaluate, and create the main themes revealed by the researcher ( Braun & Clarke, 2019; Galloway, Fred J.; Jenkins, 2005). The researcher used the NVivo 12 program to facilitate coding and categorization. It further analyzes all the codes and categories that allow the merging and even separation of codes into simpler code and can answer research questions in the main theme.

After the data is collected, then the members are checked ( Lincoln, Y.S., and Guba, 1985), which is used to check the credibility of participants. They were asked to clarify that their contributions were accurately reflected in the data. Meanwhile, the researcher also triangulated to reduce bias by cross-checking participants’ answers ( Anney, 2014). Thus, the involvement of all researchers in examining the data will support the integrity of the findings.

### Ethical considerations

The Institute for Research and Community Service at Djuanda University, West Java, Indonesia, has approved this research. The researcher also provided approval letters to all respondents. Written consent to participate from the respondent was obtained in accordance with contract document number 363.1/LPPM/K-X/X/2024. The respondent gave his consent without coercion from anyone. Furthermore, all data obtained will remain confidential to protect respondents’ rights and privacy.

## Results and Discussion

### Result

**Figure 1.**
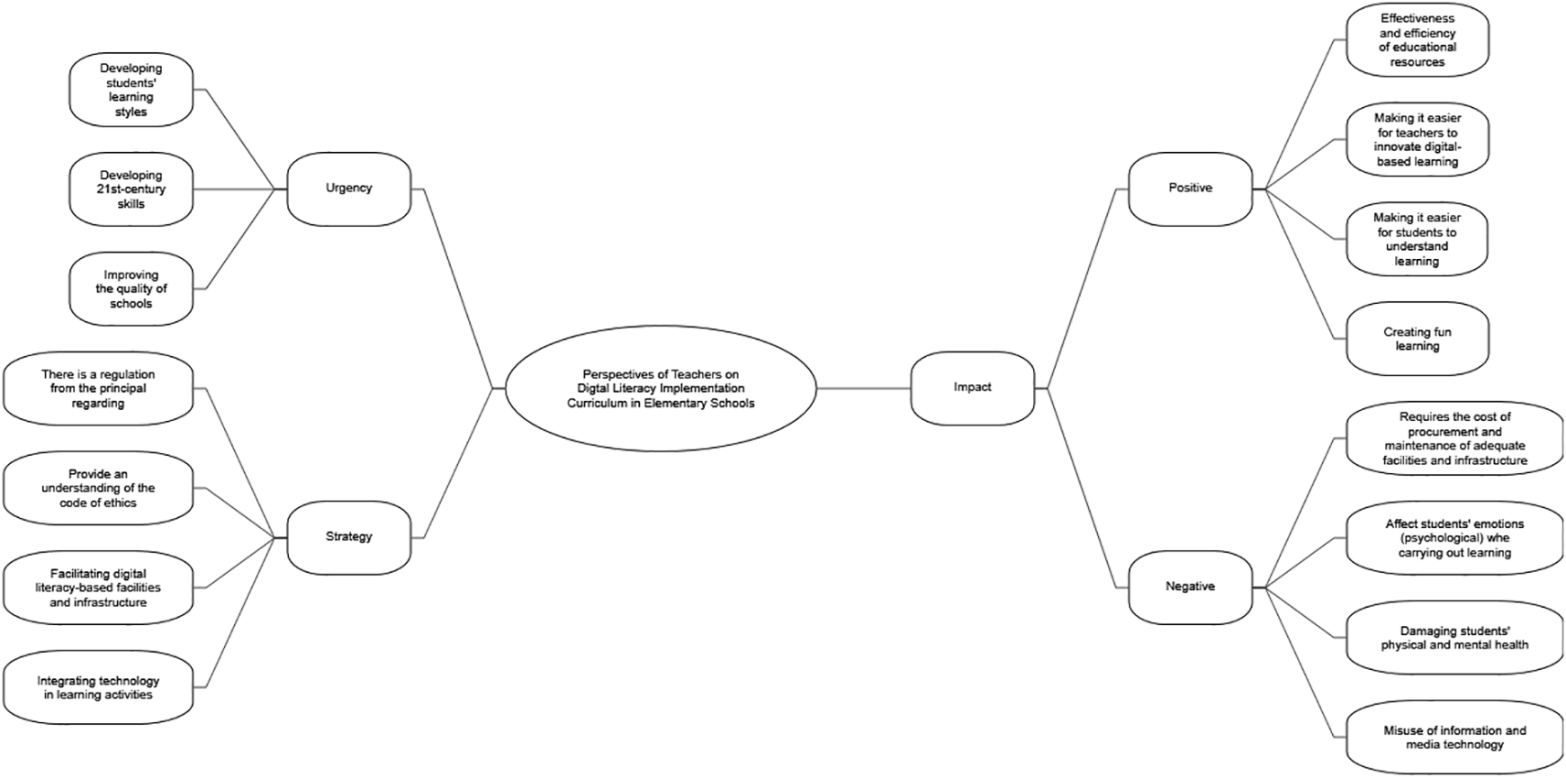
The main themes of thematic analysis (use Nvivo 12).

Thematic analysis revealed three main themes, namely (1) the urgency of implementing digital literacy in the curriculum in elementary schools, 2) teachers’ strategies in implementing digital literacy in the curriculum in elementary schools, and 3) the positive and negative impacts of the implementation of digital literacy in the curriculum in elementary schools. All themes are summarized in Figure 1.

### The urgency of digital literacy implementation curriculum in elementary schools

The three subthemes of the urgency of implementing digital literacy in the curriculum in elementary schools are to implement lifelong learning, improve school quality, and develop 21st-century skills.

The application of digital literacy can produce curriculum innovation as a demand in learning activities in the era of society 5.0. In addition, the application of digital literacy can also strengthen children’s and teachers’ understanding of the nation’s culture, which is currently declining in quality. Teacher 02 states that:

*Technological developments make it easier for everyone to communicate and interact with various countries; therefore, it is necessary to strengthen the understanding of the nation’s culture, one of which is through the application of the Pancasila student profile as a characteristic of Indonesian culture. (Teacher 02)*

Digital literacy is also necessary to improve the quality of education in elementary schools. It makes it easier for teachers to achieve the learning goals written in the learning plan to produce meaningful learning. In addition, digital literacy can also demand an increase in teachers’ competence. Teachers always conduct training to improve competence to help develop students’ talents and interests.

*Applying digital literacy in elementary schools requires teachers to develop competencies to be more professional. (Teacher 21)*

Furthermore, the application of digital literacy in elementary schools can also improve 21st-century skills, where teachers and students must explore how to think critically and creatively, solve problems, make good decisions, and be responsible. Thus, the holistic potential of students, ranging from intellectual, emotional, physical, social, aesthetic, and spiritual, is easy to achieve. Teacher 31 states that:

*One of the independent learning strategies is to facilitate students with technology-based teaching resources and learning media so that students can quickly think critically and creatively when solving problems. (Teacher 31)*

### Teachers’ strategies in digital literacy implementation curriculum in elementary schools

There are four strategic subthemes in implementing digital literacy. The first is the regulation from the principal about the rules for implementing digital literacy, facilitating infrastructure, integrating technology in learning activities, and providing an understanding of digital literacy ethics from an early age.

School principals must make regulations on the rules for implementing digital literacy so that it runs well according to the curriculum developed. These rules start from learning procedures, finances, and infrastructure facilities in the school work program. Schools also need to integrate technology into learning activities, and teachers must always use Google for Education when carrying out learning activities. When implementing digital literacy, schools also need to facilitate infrastructure ranging from the internet, laptops, networks, projectors, and infrastructure to support the implementation of digital literacy in elementary schools, smart TVs, computer laboratories, and digital libraries as learning sources and tools. Closed-circuit Television (CCTV) monitors classroom learning so teachers and principals can supervise learning activities daily. Some teachers stated:

*Students can study comfortably in the computer labs provided, and teachers can monitor their learning activities directly or through the school’s Closed-Circuit Television (CCTV) recordings. Technology has made it easier for teachers to conduct learning and assessment activities. (Teacher 35)*

Each school can have different policies regarding digital literacy. Some schools implement project-based learning, such as creating books or poems, and conduct contextual-based learning. Several other schools apply digital literacy to the extracurricular, intracurricular, and co-curricular activities of the school.

*Digital literacy is carried out through extracurricular activities at school so that students can explore various activities to develop their talents and interests in technology skills. (Teacher 03)*

To avoid misuse of technology, schools make rules that require all school residents to be able to understand digital literacy ethics, which provide information on how to behave, good manners in communicating, and respecting each other when communicating online (such as avoiding communicating with rude, insulting, and hate speech messages). In addition, the school also provides an understanding of the importance of personal account security when using mobile phones and social media. Meanwhile, teachers and parents are required to accompany students when using the internet. The teacher stated:

*Schools, in collaboration with the Ministry of Communication and Information of the Republic of Indonesia, always socialize on how to use them and the positive and negative impacts of implementing digital literacy. (Teacher 23)*

## The positive and negative impacts of the digital literacy implementation curriculum in elementary schools

### Positive impact

There are four subthemes of the positive impact of implementing digital literacy: first, the effectiveness and efficiency of educational resources; second, making it easier for teachers to innovate digital-based learning; third, making it easier for students to understand learning; and fourth, it can create fun learning.

Implementing digital literacy provides wider access to information and educational resources, helping teachers create conducive classrooms and making it easier for students to understand the subject. Students are accustomed to using technology in every learning so that it impacts increasing students’ critical skills, creativity, and collaboration. In addition, teachers can easily direct students to collaborate as a team in completing assignments. Students become more focused and critical because they can access much information through digital media.

Digital literacy also helps make it easier for teachers to innovate learning because access to information is wide open. Teachers can easily find credible references and learning resources, easily create interesting learning media and evaluation tools, so that it impacts the ease of students to improve their thinking, verbal, collaboration, and reading skills. The Master states that:

*Schools that have implemented digital literacy learning have an impact on the workforce efficiency that teachers and students must expend. The presence of technology can have an impact on teachers, saving energy and time at the same time. (Teacher 11)*

Applying digital literacy can also help schools save time and educational funds. Activities that usually require a lot of paper when conducting exams can now be completed online. Schools no longer spend money on paper, ballpoint pens, and other office stationery, so learning activities run more effectively and efficiently.

### Negative impact

The four subthemes of the negative impact of implementing digital literacy on students and schools are: first, too often using technology media can damage physical health; second, it affects children’s emotional stability; third, the misuse of information and technology media occurs; and fourth, significant costs are needed for the procurement and maintenance of infrastructure facilities.

The use of digital devices for an extended period of time can cause physical damage, ranging from visual impairments, nerve disorders, brain disorders, sleep disorders, poor posture, and problems with the back and neck. Excessive interaction with social media and online content can also increase stress and anxiety levels in students and teachers. The Master states that:

*Some students have red eyes because they use gadgets too often. (Teacher 20)*

*Teachers sometimes have trouble sleeping because they use digital devices too often at school and home to complete assignments. (Teacher 44)*

Not only that, but using digital devices can also affect emotional stability. Students and teachers who have too much workload to complete using laptops and mobile phones have the potential to develop mental disorders such as depression, anxiety, and dependence.

*My fellow teachers, if they have a lot of assignments and deadlines, sometimes they become emotionally unstable, some get angry, some always feel excessively anxious, and some like to cry alone in class. (Teacher 41)*

In addition, it is difficult for teachers and parents to provide assistance and briefings on the dangers of excessive use of technological devices, which has an impact on many elementary school students who are addicted to gadgets and access harmful content (pornographic videos) freely through mobile phones and laptops outside of school hours. In fact, many elementary school students are victims of cyberbullying because of the actions of close friends in their class. Not only that, the lack of supervision from schools and parents also impacts the number of students who spread fake news (hoaxes) through social media.

*There have been students who are addicted to gadgets because they often play games at home, and there are even students who watch pornographic videos through mobile phones. (Teacher 36)*

The application of digital literacy in elementary schools also impacts the significant school budget expenditure. Purchasing laptops, infocusts, smart TVs, and other digital facilities requires high costs. Not only that, but schools also have to pay for electricity, Wi-Fi, and maintenance throughout the year. Therefore, not all schools have complete digital learning facilities. Most elementary schools in remote villages in Indonesia do not have proper digital learning tools. Teacher 35 affirms:

*Schools must budget the cost of laptops, infocusts, and smart TVs in their school budget and learning plans. (Teacher 35)*

## Discussion

In this study, the researchers conducted an online survey and interviewed teachers who have implemented digital literacy for at least 3 years. The researcher asked questions about the strategy, urgency, and positive and negative impacts of applying digital literacy in the independent curriculum in elementary schools. Teachers’ statements were analyzed based on theoretical background and research findings related to the application of digital literacy in elementary schools. Using thematic analysis provides an overview for researchers to investigate further strategies and the positive and negative impacts of digital literacy on the independent curriculum in elementary schools.

The researcher identified several themes and subthemes that reflected teachers’ opinions on applying digital literacy. Although the Indonesian government has made policies to implement digital literacy, the limited software owned by schools ( Rahmah, 2015) has an impact on the difficulty of implementing digital literacy in elementary schools.

Therefore, **first,** a strategy is needed in implementing digital literacy in elementary schools through the creation of rules and regulations from schools regarding the use of digital literacy in accordance with the independent curriculum in elementary schools ( McGeehan & Norris, 2020) to integrate digital learning in various learning activities ( Falloon, 2020; Rahmah, 2015). Teachers must use learning media ( Davis et al., 2021; Lazonder et al., 2020) and digital-based learning assessments ( Falloon, 2020; Jin et al., 2020; Wilkes et al., 2020). Teachers must also be given competency training according to the times ( List et al., 2020), to be able to educate and guide students using technology ( Falloon, 2020) and be able to make a learning plan ( Rasmitadila et al., 2020).

In addition, to be able to implement digital literacy learning in elementary schools properly, support is needed from all education stakeholders, ranging from principals, teachers, parents, the government, and even the involvement of universities in providing holistic assistance ( Aliyyah et al., 2023; Rasmitadila et al., 2020). Digital literacy in elementary schools requires teachers to be proficient in using technology to build character, gain new knowledge, and avoid hoax information ( Rahmah, 2015; Tavares et al., 2023).

**Second,** the implementation of good digital literacy has an impact on the ease of teachers finding learning resources ( Falloon, 2020) and teaching materials ( Aliyyah et al., 2024) quickly, resulting in meaningful learning ( Condie & Pomerantz, 2020) together with students. The many media, methods, and digital-based learning models that teachers can use in learning activities will make it easier for students to develop skills according to their talents and interests ( Csernoch & Biró, 2015; Lee et al., 2018; Park et al., 2021). The higher the quality of teacher competence ( Falloon, 2020; Shafie et al., 2019) in schools, the more it can further improve the quality of education ( Hvidston et al., 2019; Kesici & Ceylan, 2020).

**Third,** the application of digital literacy in elementary schools has a positive impact on the development of critical thinking ( Davis et al., 2021), creativeness ( Tavares et al., 2023; Van Laar et al., 2017), Innovation ( Falloon, 2020), problem-solving skills ( Van Laar et al., 2017) and team collaboration skills ( Bett, 2016; Smeaton & Callan, 2005) which is a 21st century skill prowess ( Güneş & Bahçivan, 2018; Van Laar et al., 2017).

To overcome the negative impact of the implementation of digital literacy learning, schools can also provide education consistently to include moral values in the curriculum and subjects ( Lynch et al., 2017), provide education on the importance of communication ethics through online media ( Fourie, 2017) and assist so that students always maintain their privacy accounts to be protected from fraud, and online crime ( Pyrooz et al., 2015; Stalans & Finn, 2016).

Schools must also assist with the procedures for using technological devices ( Larsson, Ranada, & Lidström, 2019). Starting from the use of the night mode feature or blue light filter ( Luqman et al., 2021), take regular breaks by stretching your body and taking your eyes off the screen of your laptop or mobile phone, and provide information about the wise use of technological tools ( Stratigea et al., 2015).

Not only that, but in order to anticipate the negative impact of digital use ( Saputra & Al Siddiq, 2020), Schools also need to provide regular assistance to parents and teachers through parenting activities ( Smith, 2010; Vincent, 2017). Good cooperation between schools and parents impacts the easy achievement of learning objectives and school curriculum.

### Limitation

The limitation of this study lies in determining the participant criteria that require elementary school teachers who have carried out digital literacy learning activities for at least three years. Meanwhile, not all elementary school teachers can use digital literacy-based learning, so the number of participants is still limited. The research was also only conducted on elementary school units, so it has not been carried out in junior high school and Senior High School.

## Conclusion

The implementation of digital literacy is very important because it impacts the quality of education, the efficiency and effectiveness of school resource use, the development of school curriculum, the development of teacher competencies through digital platforms, and the development of 21st-century skills in students.

To implement digital literacy properly in accordance with the school curriculum, regulations are needed that regulate the availability of adequate facilities and infrastructure, integrate technology in learning activities, and have a code of ethics that is used as learning rules for teachers and students.

The researcher recommends the results of this study to elementary schools so that they can sustainably implement digital literacy and are prepared based on the curriculum according to the school’s work plan. The researcher also recommends that the government make policies on the rules for implementing digital literacy in elementary schools. In addition, The future research could be research in building the model of digital literacy-based curriculum.

## Data Availability

Figshare: ‘Perspectives of Teachers on Digital Literacy Implementation Curriculum in Elementary Schools’ Doi: 10.6084/m9.figshare.30393514 ( Aliyyah et al., 2025). This project contains the following underlying data:
• Data sheet and Figure 1. The main themes of thematic analysis (use Nvivo 12) Data are available under the terms of the Creative Commons Attribution 4.0 International license (CC-BY 4.0).
