# Perspectives of Teachers on Digital Literacy Implementation Curriculum in Elementary Schools

**DOI:** 10.12688/f1000research.172273.3

**Published:** 2026-07-03

**Authors:** Rusi Rusmiati Aliyyah, Teguh Prasetyo, Widyasari Widyasari

**Affiliations:** 1 Faculty of Teacher Training and Education, Universitas Djuanda, Bogor, West Java, 16720, Indonesia; 2 Faculty of Educational Technology, Universitas Ibn Khaldun, Bogor, West Java, Indonesia

**Keywords:** Curriculum Management, Digital Literacy, Elementary School, Thematic analysis, 21st century skills

## Abstract

The development of a digital literacy-based curriculum in elementary schools is an independent, student-centered approach to learning through the effective and efficient use of digital technology. The research aims to explore teachers’ perceptions of implementing and development a digital literacy-based curriculum to improve learning quality in primary schools. This research uses a quasi-qualitative approach. Data were collected through semi-structured interviews with 48 teachers from 26 primary schools in seven provinces in Indonesia, while data analysis used thematic analysis. The research results summarize the statements of elementary school teachers and reveal three main themes: the urgency, strategies, and positive and negative impacts of implementing and development a digital literacy-based curriculum in elementary schools. Participants stated that implementing and development a digital literacy-based curriculum is very important because it is carried out through the management of a good strategy to analyze positive and negative impacts in order to create a learning system that is adaptive to technological developments, improve students’ digital competence, and help develop 21st century skills. This research concludes that elementary schools need to manage and develop curricula, prepare regulations governing the use and availability of infrastructure to implement digital literacy, integrate technology into learning activities, and provide an understanding of digital ethics. The research recommends that elementary schools implement a digital literacy-based curriculum continuously, integrated, and collaborating with universities to assist in curriculum management and the Indonesian government needs to make a digital literacy-based curriculum development policy so that elementary schools have a clear and measurable barometer in managing the curriculum.

## Introduction

Digital literacy is the knowledge and skills of consumers in utilizing digital media ( Lazonder et al., 2020) to develop curriculum and upskill in the school environment to support students’ cognitive, social, and emotional abilities ( Churchill, 2020; Jang et al., 2021; UNESCO Institute for Statistics, 2018; Van Laar et al., 2017). Information and Communication Technology (ICT)is an example of digital-based childhood socialization that can expand communication, interpersonal relationships, and students’ thinking skills ( Van Laar et al., 2017). Nevertheless, digital literacy includes not only technical skills in using digital devices and applications, but also a deep understanding of ethics, security, and positive engagement in the online world ( Jang et al., 2021). The development of a digital literacy-based curriculum in elementary schools plays an important role in building the foundation of digital literacy for children ( Purnama et al., 2021). Developing a digital literacy-based curriculum is essential to enhance 21st century learning ( Churchill, 2020). Research states that digital literacy learning has an impact on 21st-century skills development ( Churchill, 2020; Kim et al., 2014; List, 2019; Purnama et al., 2021; Siddiq et al., 2017) that focuses on competencies so that students have critical thinking skills and good metacognition ( Siddiq et al., 2017).

Various countries have implemented digital literacy learning, which is packaged in the development of a curriculum in accordance with the potential of schools to improve the quality of education in their countries. Selected European countries (Slovakia, Czechia, Poland, the United Kingdom, and Ireland), as well as the United States and Sweden, include digital literacy in primary school students’ learning to improve students’ numeracy literacy skills ( Javorský & Horváth, 2014; UNESCO Institute for Statistics, 2018). The results of the study stated that European countries use six national educational technology standards in basic education, namely creativity and innovation, communication and collaboration, research and information fluency, critical thinking, problem solving, decision making, technology operations and concepts ( Javorský & Horváth, 2014). China has developed a digital learning curriculum to improve the literacy skills of grade 4 elementary school students ( Harvey & Brooks, 2022). Hungary develops a curriculum to improve information literacy skills and competencies ( Varga & Egervári, 2014). Canada designs classroom-based cybersecurity, privacy, and digital literacy games for elementary school students ( Maqsood & Chiasson, 2021). The United States is developing a digital model to make it easier for elementary school teachers to implement technology-based curriculum ( Obel-Omia, 2016). Taiwan develops inquiry and six-frame learning to integrate information literacy into the primary school curriculum ( Chen, 2018). Meanwhile, Korea developed a national curriculum that teaches software learning in the private activities of 5-year-olds ( Lee et al., 2018).

Meanwhile, to improve the quality of elementary schools, especially in developing a digital literacy based curriculum, Indonesia has formed the school literacy movement as a comprehensive effort that involves school residents (teachers, students, and parents of students) and the community to participate in the education ecosystem. Meanwhile, to answer the demands of technology-based 21st century learning, in 2021, the Ministry of Education, Culture, Research, and Technology of the Republic of Indonesia launched a digital literacy module in elementary schools that contains strategies for implementing digital literacy carried out inside and outside the classroom in the form of extracurricular activities with a learning focus on time management, cyberbullying, cybersecurity management, Privacy Management, Critical Thinking, and Digital Empathy ( Rahmah, 2015). Indonesia has also developed a digital corner for technology-based learning activities in all educational units, including elementary schools, it has also developed computational thinking in the curriculum of local school content ( Aliyyah et al., 2024). All of these digital literacy-based learning activities are contained in the curriculum developed by the Indonesian government under the name of the merdeka curriculum. Merdeka curriculum is a curriculum with diverse intracurricular learning with more optimal content so that students have enough time to explore concepts and strengthen competencies. Merdeka curriculum encourages the mastery of digital skills, where students learn to use technology wisely, safely, and critically with the information they encounter ( Emawati et al., 2024). Merdeka curriculum is a curriculum that has been implemented in Indonesia since February 2022 with a diverse and optimal intracurricular learning design so that students have enough time to explore concepts and strengthen competencies ( Ministry of Education and Culture of the Republic of Indonesia, 2022). Merdeka curriculum provides flexibility for educational units and teachers in designing learning that is in accordance with the learning context and needs and student-centered ( Aliyyah et al., 2023). In addition, merdeka curriculum emphasizes the importance of utilizing various sources of knowledge, not only textbooks but also information obtained through digital platforms and other related references ( Emawati et al., 2024).

However, elementary schools in Indonesia have difficulty implementing government programs due to various challenges. Among them are the limited software owned by the school, the low awareness of users in utilizing the facilities owned by them ( Ministry of Education, Culture, 2021), low public awareness and knowledge about Information and Communication Technologies (ICT) ( Rasmitadila et al., 2020), there are no special assistance for teachers and students after the elimination of special subjects on ICT in the elementary school curriculum so that teachers and students are not given knowledge and understanding about the ethics of using ICT ( Aliyyah et al., 2023). In addition, the lack of adequate technological facilities and infrastructure in schools affects the limited ability of teachers and students to use technology ( Rahmah, 2015). Based on these problems, this research is important to answer and provide alternative solutions to problems that occur in Indonesia.

The *state-of-the-art* in this study was conducted using the VOSviewer system, with data sourced from the Scopus database. The researcher included 13 keywords: *digital literacy, technology, information, school, research, student, education, elementary school, child, study, teacher, curriculum, literacy*, and *child*, covering the last 10 years (2014-2024). There are 50 articles related to keywords, and research on the implementation of a digital literacy-based curriculum in elementary schools has only been carried out since 2020, and has not been widely done in the world, nor has it ever been conducted on the theme of teacher perception models about the implementation of a digital literacy-based curriculum in elementary schools.

The novelty of this research is to produce a model of teachers’ perception of the implementation of digital literacy-based curriculum development through the theme of urgency, strategies and positive and negative impacts on learning so that it can be a reference for elementary schools and the government in implementing and developing the curriculum to improve the quality of education in Indonesia.

This research aims to explore teachers’ opinions regarding the implementation of digital literacy-based curriculum in elementary schools in Indonesia. Three main questions are the theme of this study. The three questions are:

1. The urgency of implementing a digital literacy-based curriculum in elementary schools?
2. What is the teacher’s strategy in implementing a digital literacy-based curriculum in elementary schools?
3. What are the positive and negative impacts of the implementation of a digital literacy-based curriculum in elementary schools?
  The urgency of conducting this research can provide an overview of the development of a digital literacy-based curriculum in elementary schools. Meanwhile, the strategy can provide direction and focus for implementing a digital literacy-based curriculum in elementary schools. Positive impacts can result from implementing a digital literacy-based curriculum in elementary schools. Meanwhile, negative impacts can explain anticipation and efforts to prevent the risk of errors in the implementation and development of digital literacy-based curriculum in elementary schools.

## Methodology

### Research design

The approach used in this research was quasi-qualitative with a simple research design. Quasi-qualitative research is a study with the primary objective of objectively describing a situation according to the problem ( Cropley, 2019). Meanwhile, according to Bungin (2020), quasi-qualitative is a part of research influenced by positivism, which is used in the presentation of theory, a kind of deductive approach, so this research cannot be entirely qualitative. This can be seen during data analysis. Quasi-qualitative research is suitable for describing the lives of information sources. One type of quasi-qualitative research is a simple research design (SRD). SRD is a research design used by a researcher to reflect on findings in the field by using theory to solve the problems encountered ( Bungin, 2020). The research procedure of SRD was carried out with five main steps, namely (1) Selecting the social context and determining the research question (Social context and research question); (2) Conducting a literature review (Literature Review); (3) Conducting research methods and collecting data (Research methods and data collection); (4) Analyzing data (Data Analysis); (5) Reporting research results (Reporting) ( Bungin, 2020).

### Participants

The participants in this study were 48 teachers spread across 26 elementary schools in seven provinces in Indonesia, covering the regions of West Java, Jakarta, Central Java, Yogyakarta, East Java, North Sumatra, and South Kalimantan. This is in accordance with Creswell’s opinion about the concept of qualitative research ( Creswell, J. W., & Poth, 2018). The criteria for selecting elementary school teachers are based on experience implementing digital literacy in elementary schools for at least 3 years. Teacher data were taken from seven provinces, based on data from the Ministry of Education and Culture of the Republic of Indonesia, which states that these seven regions have the highest levels of digital literacy in Indonesia in 2022 and 2023. The sampling technique aims to be used by conducting direct interviews with teachers who have used digital-based learning media in the Form of the internet, PowerPoint, YouTube, e-books, learning videos, learning applications, learning games, Google Classroom, Moodle, online learning multimedia, and others that support digital-based learning both inside and outside the classroom at least four times a week. Descriptive data on demographic characteristics, including gender, age, length of teaching, and level of education, are presented in Table 1.

**Table 1.** Participant’s profiles.

Respondent profile	Frequency	Served (%)
Gender		
Woman	32	67%
Man	16	33%
Age		
20-29	9	19%
30-39	20	42%
40-49	9	19%
50-59	10	20%
Education level		
Bachelor	43	90%
Magister	5	10%
Doctor	0	0%
Long teaching time		
1-5 years	13	27%
6-10 years	3	6%
11-15 years	8	17%
16-20 years	10	21%
21-25 years	8	17%
Over 25 years old	6	12%

### Data collection

Data were collected through in-depth interviews with 48 teachers across 26 elementary schools in seven provinces in Indonesia, according to the set criteria. The interview guide is based on the concepts of exploration and meaning-making regarding the implementation of a digital literacy-based curriculum in elementary schools. The three aspects asked about in the interview were: urgency, strategies, and the positive and negative impacts of implementing a digital literacy-based curriculum.

The semi-structured interview was conducted over 3 months, from August to October 2024, for 1–2 hours with 48 respondents via Zoom. The researcher conducted daily interviews with 1 respondent on the effective (work) day of school on Monday-Thursday. Before the interview began, the researcher conveyed to the respondents that their answers would be kept confidential. Furthermore, the interview results were written, and transcripts were prepared for each respondent to create an initial code based on the themes’ similarities using NVivo 12.

In the first stage, the researcher involved principals in elementary schools who implemented digital literacy in 7 pre-selected provinces. The principal provides a Google Form link containing questions to the teacher as the respondent, along with the criteria for completing the Form. After the data were entered and the initial coding was completed, the researchers selected five respondents from the 48 respondents for in-depth interviews to clarify and confirm answers not found in Google Forms. The selection of the five respondents was based on the results of examining the respondents’ answers in Google Forms, using the most detailed answer criteria for the questions and research objectives.

### Instruments

The instrument used in this study is in the in-depth interviews which are then made in the form of transcripts containing teachers’ ideas or opinions about the application of digital literacy in the curriculum in elementary schools based on their experience so far. The following are the questions given to teachers:

1. Explain the urgency of implementing a digital literacy-based curriculum in elementary schools!
2. Explain teachers’ strategies in the implementation of a digital literacy-based curriculum in elementary schools!
3. Explain the positive and negative impacts of the implementation of a digital literacy-based curriculum in elementary schools!

### Data analysis

The data analysis used was a thematic analysis technique to identify, evaluate, and create the main themes revealed by the researcher ( Braun & Clarke, 2019; Galloway & Jenkins, 2005). The researcher used the NVivo 12 program to facilitate coding and categorization. It further analyzes all the codes and categories that allow the merging and even separation of codes into simpler code and can answer research questions in the main theme.

After the data is collected, then the members are checked ( Lincoln, Y.S., and Guba, 1985), which is used to check the credibility of participants. They were asked to clarify that their contributions were accurately reflected in the data. Meanwhile, the researcher also triangulated to reduce bias by cross-checking participants’ answers ( Anney, 2014). Thus, the involvement of all researchers in examining the data will support the integrity of the findings.

### Ethical considerations

The Institute for Research and Community Service at Djuanda University, West Java, Indonesia, has approved this research. The researcher also provided approval letters to all respondents. Written consent to participate from the respondent was obtained in accordance with contract document number 363.1/LPPM/K-X/X/2024. The respondent gave his consent without coercion from anyone. Furthermore, all data obtained will remain confidential to protect respondents’ rights and privacy.

## Results and Discussion

### Result

**Figure 1.**
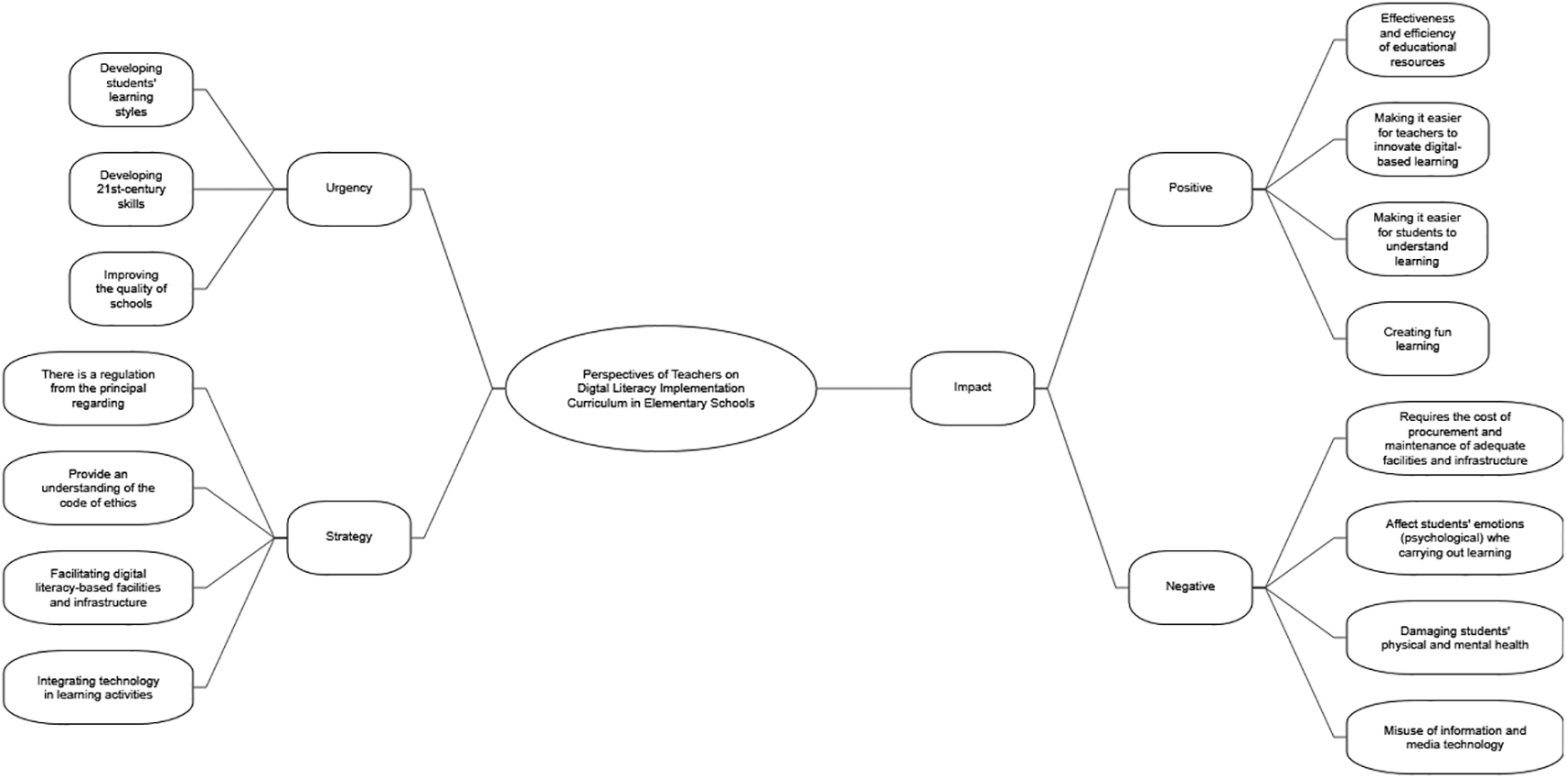
The main themes of thematic analysis (use Nvivo 12).

The thematic analysis revealed three main themes, namely (1) the urgency of implementing a digital literacy-based curriculum in elementary schools, (2) teachers’ strategies in implementing a digital literacy-based curriculum in elementary schools, and (3) the positive and negative impacts of implementing a digital literacy-based curriculum in elementary schools. All themes are summarized in Figure 1.

### The urgency of implementing a digital literacy-based curriculum in elementary schools

The three subthemes regarding the urgency of implementing a digital literacy based curriculum in elementary schools are to realize lifelong learning, improve the quality of schools, and develop 21st-century skills.

The application of digital literacy can produce curriculum innovation as a demand in learning activities in the era of society 5.0. In addition, the application of digital literacy can also strengthen children’s and teachers’ understanding of the nation’s culture, which is currently declining in quality. Teacher 02 states that:

*Technological developments make it easier for everyone to communicate and interact with various countries; therefore, it is necessary to strengthen the understanding of the nation’s culture, one of which is through the application of the Pancasila student profile as a characteristic of Indonesian culture. (Teacher 02)*

Digital literacy is also necessary to improve the quality of education in elementary schools. It makes it easier for teachers to achieve the learning goals written in the learning plan to produce meaningful learning. In addition, digital literacy can also demand an increase in teachers’ competence. Teachers always conduct training to improve competence to help develop students’ talents and interests.

*Applying digital literacy in elementary schools requires teachers to develop competencies to be more professional. (Teacher 21)*

Furthermore, the application of digital literacy in elementary schools can also improve 21st-century skills, where teachers and students must explore how to think critically and creatively, solve problems, make good decisions, and be responsible. Thus, the holistic potential of students, ranging from intellectual, emotional, physical, social, aesthetic, and spiritual, is easy to achieve. Teacher 31 states that:

*One of the independent learning strategies is to facilitate students with technology-based teaching resources and learning media so that students can quickly think critically and creatively when solving problems. (Teacher 31)*

### Teachers’ strategies in the implementation of digital literacy-based curriculum in elementary schools

There are four strategic subthemes in the implementation of a digital literacy-based curriculum. The first is the regulation from the principal about the rules for implementing digital literacy, facilitating infrastructure, integrating technology in learning activities, and providing an understanding of digital literacy ethics from an early age.

School principals must make regulations on the rules for implementing digital literacy so that it runs well according to the curriculum developed. These rules start from learning procedures, finances, and infrastructure facilities in the school work program. Schools also need to integrate technology into learning activities, and teachers must always use Google for Education when carrying out learning activities. When implementing digital literacy, schools also need to facilitate infrastructure ranging from the internet, laptops, networks, projectors, and infrastructure to support the implementation of digital literacy in elementary schools, smart TVs, computer laboratories, and digital libraries as learning sources and tools. Closed-circuit Television (CCTV) monitors classroom learning so teachers and principals can supervise learning activities daily. Some teachers stated:

*Students can study comfortably in the computer labs provided, and teachers can monitor their learning activities directly or through the school’s Closed-Circuit Television (CCTV) recordings. Technology has made it easier for teachers to conduct learning and assessment activities. (Teacher 35)*

Each school can have different policies regarding digital literacy. Some schools implement project-based learning, such as creating books or poems, and conduct contextual-based learning. Several other schools apply digital literacy to the extracurricular, intracurricular, and co-curricular activities of the school.

*Digital literacy is carried out through extracurricular activities at school so that students can explore various activities to develop their talents and interests in technology skills. (Teacher 03)*

To avoid misuse of technology, schools make rules that require all school residents to be able to understand digital literacy ethics, which provide information on how to behave, good manners in communicating, and respecting each other when communicating online (such as avoiding communicating with rude, insulting, and hate speech messages). In addition, the school also provides an understanding of the importance of personal account security when using mobile phones and social media. Meanwhile, teachers and parents are required to accompany students when using the internet. The teacher stated:

*Schools, in collaboration with the Ministry of Communication and Information of the Republic of Indonesia, always socialize on how to use them and the positive and negative impacts of implementing digital literacy. (Teacher 23)*

## The positive and negative impacts of the implementation of a digital literacy-based curriculum in elementary schools

### Positive impact

There are four subthemes of positive impact from the implementation of a digital literacy-based curriculum: first, the effectiveness and efficiency of educational resources; second, making it easier for teachers to innovate digital-based learning; third, making it easier for students to understand learning; and fourth, it can create fun learning.

Implementing digital literacy provides wider access to information and educational resources, helping teachers create conducive classrooms and making it easier for students to understand the subject. Students are accustomed to using technology in every learning so that it impacts increasing students’ critical skills, creativity, and collaboration. In addition, teachers can easily direct students to collaborate as a team in completing assignments. Students become more focused and critical because they can access much information through digital media.

Digital literacy also helps make it easier for teachers to innovate learning because access to information is wide open. Teachers can easily find credible references and learning resources, easily create interesting learning media and evaluation tools, so that it impacts the ease of students to improve their thinking, verbal, collaboration, and reading skills. The Master states that:

*Schools that have implemented digital literacy learning have an impact on the workforce efficiency that teachers and students must expend. The presence of technology can have an impact on teachers, saving energy and time at the same time. (Teacher 11)*

Applying digital literacy can also help schools save time and educational funds. Activities that usually require a lot of paper when conducting exams can now be completed online. Schools no longer spend money on paper, ballpoint pens, and other office stationery, so learning activities run more effectively and efficiently.

### Negative impact

The four subthemes of the negative impact of the implementation of a digital literacy-based curriculum on students and schools are: first, too often using technology media can damage physical health; second, it affects children’s emotional stability; third, the misuse of information and technology media occurs; and fourth, significant costs are needed for the procurement and maintenance of infrastructure facilities.

The use of digital devices for an extended period of time can cause physical damage, ranging from visual impairments, nerve disorders, brain disorders, sleep disorders, poor posture, and problems with the back and neck. Excessive interaction with social media and online content can also increase stress and anxiety levels in students and teachers. The Master states that:

*Some students have red eyes because they use gadgets too often. (Teacher 20)*

*Teachers sometimes have trouble sleeping because they use digital devices too often at school and home to complete assignments. (Teacher 44)*

Not only that, but using digital devices can also affect emotional stability. Students and teachers who have too much workload to complete using laptops and mobile phones have the potential to develop mental disorders such as depression, anxiety, and dependence.

*My fellow teachers, if they have a lot of assignments and deadlines, sometimes they become emotionally unstable, some get angry, some always feel excessively anxious, and some like to cry alone in class. (Teacher 41)*

In addition, it is difficult for teachers and parents to provide assistance and briefings on the dangers of excessive use of technological devices, which has an impact on many elementary school students who are addicted to gadgets and access harmful content (pornographic videos) freely through mobile phones and laptops outside of school hours. In fact, many elementary school students are victims of cyberbullying because of the actions of close friends in their class. Not only that, the lack of supervision from schools and parents also impacts the number of students who spread fake news (hoaxes) through social media.

*There have been students who are addicted to gadgets because they often play games at home, and there are even students who watch pornographic videos through mobile phones. (Teacher 36)*

The application of digital literacy in elementary schools also impacts the significant school budget expenditure. Purchasing laptops, infocusts, smart TVs, and other digital facilities requires high costs. Not only that, but schools also have to pay for electricity, Wi-Fi, and maintenance throughout the year. Therefore, not all schools have complete digital learning facilities. Most elementary schools in remote villages in Indonesia do not have proper digital learning tools. Teacher 35 affirms:

*Schools must budget the cost of laptops, infocusts, and smart TVs in their school budget and learning plans. (Teacher 35)*

## Discussion

In this study, the researchers conducted an online survey and interviewed teachers who have implemented digital literacy for at least 3 years. The researcher asked questions about the strategy, urgency, and positive and negative impacts of applying digital literacy in the independent curriculum in elementary schools. Teachers’ statements were analyzed based on theoretical background and research findings related to the application of digital literacy in elementary schools. Using thematic analysis provides an overview for researchers to investigate further strategies and the positive and negative impacts of digital literacy on the independent curriculum in elementary schools.

The researcher identified several themes and subthemes that reflected teachers’ opinions on applying digital literacy. Although the Indonesian government has made policies to implement digital literacy, but the limited software owned by schools ( Rahmah, 2015) has an impact on the difficulty of implementing digital literacy in elementary schools. This emphasizes that the policies that have been made by the government have not been maximized and concrete steps are needed to be able to implement curriculum development in elementary schools.

Therefore, **first**, a **strategy** is needed to implement a digital literacy-based curriculum in elementary schools. This can be done through: 1) Making rules and regulations for digital literacy use that align with the independent curriculum ( McGeehan & Norris, 2020). These should integrate digital learning across various activities and foster understanding of digital ethics ( Falloon, 2020; Rahmah, 2015). 2) Teachers should use innovative and contextual digital learning media ( Davis et al., 2021; Lazonder et al., 2020) and also provide digital-based learning assessments ( Falloon, 2020; Jin et al., 2020; Wilkes et al., 2020). 3) Teachers need ongoing competency training ( List et al., 2020), Training helps them educate and guide students using technology ( Falloon, 2020) and develop digital-based learning plans ( Rasmitadila et al., 2020).

Implementing a digital literacy-based curriculum requires teachers to be proficient in using technology. This helps build character, acquire new knowledge, and avoid misinformation ( Rahmah, 2015), this is in accordance with the concept of curriculum development applied to elementary schools ( Tavares et al., 2023).

**Second**, implementing a digital literacy-based curriculum is **urgent**. It can develop critical thinking ( Davis et al., 2021), creativity ( Tavares et al., 2023; Van Laar et al., 2017), innovation ( Falloon, 2020), problem-solving skills ( Van Laar et al., 2017) and team collaboration skills ( Bett, 2016; Smeaton & Callan, 2005). These skills make it easier for teachers to develop 21st-century competencies ( Güneş & Bahçivan, 2018). This happens because teachers can develop a learning approach according to students’ learning styles, thereby producing quality graduates according to the direction of curriculum development in the 5.0 era ( Van Laar et al., 2017).

**Third**, the implementation of a digital literacy-based curriculum has a **positive impact** on making it easier for teachers to find learning resources ( Falloon, 2020) and teaching materials ( Aliyyah et al., 2024). This leads to meaningful and enjoyable learning, as students can more easily understand the material ( Condie & Pomerantz, 2020). Teachers who innovate with digital media, methods, and learning models help students develop competencies aligned with their talents and interests ( Csernoch & Biró, 2015; Lee et al., 2018; Park et al., 2021). Better teacher competence ( Falloon, 2020; Shafie et al., 2019) so it is easier to improve the quality of education according to the goals of the Sustainable Development Goals (SDGs) ( Hvidston et al., 2019; Kesici & Ceylan, 2020).

However, because implementing a digital literacy-based curriculum can have **negative impacts**, schools must act carefully. They need to provide digital literacy learning that includes moral values. This prevents the abuse of information and technology ( Lynch et al., 2017), schools should educate students about online communication ethics ( Fourie, 2017) and help students keep accounts private to avoid online fraud and crime ( Pyrooz et al., 2015; Stalans & Finn, 2016).

Schools must provide instruction on the use of technological devices ( Larsson, Ranada, & Lidström, 2019). They should also advise on using features like night mode or blue light filters, regular breaks and guidance on device use help protect physical and mental health ( Stratigea et al., 2015). This gives an indication of the contradiction with the positive impact of the implementation of digital literacy-based curriculum development in elementary schools ( Luqman et al., 2021).

Schools should also address the emotional and psychological impact of digital use on students during learning ( Saputra & Al Siddiq, 2020). To do this, they need to provide regular support for parents and teachers with scheduled parenting activities ( Smith, 2010; Vincent, 2017). Good cooperation between schools and parents makes achieving learning goals and implementing the curriculum easier.

It is very important to implement a digital literacy-based curriculum in elementary schools. **S**upport from all education stakeholders is needed, including principals, teachers, parents, the government, and universities. University involvement can help provide curriculum assistance, resulting in more competitive teachers ( Aliyyah et al., 2023; Rasmitadila et al., 2020). An innovative curriculum can help the Indonesian government improve education quality, especially in elementary schools.

### Limitation

The limitation of this study lies in the selection of participants, who must be elementary school teachers who have conducted digital literacy learning activities for at least 3 years. Meanwhile, not all elementary school teachers can use digital literacy-based learning, so the number of participants is still limited. This research was also conducted only in elementary school units, not in junior high or high schools. Further research is expected to yield results and information on the long-term impact of implementing a digital literacy-based curriculum on student learning outcomes.

## Conclusion

The implementation of a digital literacy-based curriculum is very urgent because it **impacts** the quality of learning, the efficiency and effectiveness of school resource use, the development of teacher competencies through digital platforms, the creation of fun learning, and the development of 21st-century competencies in students. However, the implementation of digital literacy also increases the budget schools must prepare, as well as the potential for misuse of technological media, which can result in psychological disturbances in student learning.

Therefore, education units need the right **strategy** to implement and develop a digital literacy-based curriculum in elementary schools. This can be done by creating regulations governing the use and availability of adequate digital facilities and infrastructure, integrating technology into learning activities, and socializing and emphasizing the importance of digital ethics to all stakeholders so they can understand the negative impact of implementing digital literacy in elementary schools.

Implementing and develop a digital literacy-based curriculum is **very urgent and essential** because it is a demand that elementary schools must meet to remain competitive in the era of globalization. Through digital literacy-based learning, teachers can develop learning styles tailored to students’ competencies, enabling the easy optimization of 21st-century skills and ensuring learning is contextual and aligned with students’ real lives.

Based on the results of the study, the implication of this research is to provide suggestions to elementary schools to be able to implement and develop a digital literacy-based curriculum in a continuous, contextual, and integrated manner in all subjects in order to produce the quality of graduates who have character, digital ethics, and 21st-century abilities. Schools can collaborate with universities to provide assistance and develop a digital literacy-based curriculum. The researcher also recommends that the Indonesian government develop a policy on implementing a digital literacy-based curriculum in elementary schools, integrating it into all learning and providing continuous teacher training to ensure quality education, especially in the development of the curriculum.

## Data Availability

Figshare: ‘Perspectives of Teachers on Digital Literacy Implementation Curriculum in Elementary Schools’ Doi: 10.6084/m9.figshare.30393514 ( Aliyyah et al., 2025). This project contains the following underlying data:
• Data sheet and Figure 1. The main themes of thematic analysis (use Nvivo 12) Data are available under the terms of the Creative Commons Attribution 4.0 International license (CC-BY 4.0).
