# Supplementary material for: Perspectives of Teachers on Digital Literacy Implementation Curriculum in Elementary Schools
Source: F1000Res. 2026 Jul 3;14:1299. Originally published 2025 Nov 24. [Version 3] doi: 10.12688/f1000research.172273.3 (PMC12789856; doi:10.12688/f1000research.172273.3)
Supplement: Peer review history

# Reviewer report: Reviewer response for version 2

**DOI:** 10.5256/f1000research.200592.r490721

**Published:** 2026-06-10

**Authors:** Sri Marmoah

**Affiliations:** 1 Universitas Sebelas Maret, Surakarta, Central Java, Indonesia

Thank you for your efforts in revising the manuscript based on the reviewers’ comments. The revised version demonstrates substantial improvement; however, several issues still need to be addressed before the manuscript is ready for indexing.

 First, in the abstract section, the findings are not yet comprehensively presented. The authors state that three themes emerged from the study, yet the abstract only elaborates on the importance of digital literacy and does not adequately describe the other two themes. To provide a balanced overview of the findings, all three themes should be briefly summarized. In addition, the recommendation presented at the end of the abstract is unnecessarily lengthy and delivered in two separate sentences. I recommend condensing it into a single concise concluding sentence.

 Regarding the introduction, the authors have made considerable efforts to address the previous comments, and this section has improved significantly. Nevertheless, the literature review on the Merdeka Curriculum presented after the introduction appears somewhat disconnected from the overall structure of the manuscript. If the authors intend to retain a separate literature review section, they should provide theoretical discussions for all key variables, not solely the Merdeka Curriculum. Alternatively, if a standalone literature review section is not essential, the discussion of the Merdeka Curriculum could be integrated more effectively into the introduction.

 In the methods section, there appears to be an inconsistency regarding the data collection technique. The manuscript states that “data were collected through in-depth interviews,” whereas the instrument section describes the use of open-ended questions. Therefore, further clarification is needed regarding the research instruments, interview protocols, and the procedures used to collect the data.

 In the results section, the thematic analysis figure should be revised to improve its readability and visual clarity. Furthermore, the findings have not been sufficiently analyzed in relation to previous studies. The discussion should more explicitly compare the current findings with existing literature and explain whether they support, extend, or contradict earlier research.

 Finally, the conclusion section does not adequately address the third research question concerning the positive and negative impacts identified in the study. The authors should ensure that the conclusion directly answers all research questions and clearly summarizes the key positive and negative impacts revealed by the findings.

 The manuscript has improved considerably; however, the issues outlined above should be addressed to strengthen its clarity, coherence, and scholarly contribution prior to indexing.

Is the work clearly and accurately presented and does it cite the current literature?

Yes

If applicable, is the statistical analysis and its interpretation appropriate?

Not applicable

Are all the source data underlying the results available to ensure full reproducibility?

Partly

Is the study design appropriate and is the work technically sound?

Partly

Are the conclusions drawn adequately supported by the results?

Partly

Are sufficient details of methods and analysis provided to allow replication by others?

No

Reviewer Expertise:

Educational management, elementary school

I confirm that I have read this submission and believe that I have an appropriate level of expertise to confirm that it is of an acceptable scientific standard, however I have significant reservations, as outlined above.

# Response

**Published:** 2026-06-24

**Authors:** Rusi Rusmiati Aliyyah

**Affiliations:** PGSD, Universitas Djuanda, Bogor, West Java, Indonesia

Dear

 Prof. Sri Marmoah

 Attached is the revised article based on the suggestions and input provided.

 The article uploaded is a revised article based on input from reviewer F1000Research so that it has a better quality than the previous article (versi 2). The difference between this article (version 3) and the previous article (version 2) lies in the abstract section that has been sharpened with three research themes and recommendations that have been refined to be more concise. The author also integrates the concept of Merdeka curriculum in Indonesia in the introduction. In the methodology section, the author emphasizes the data collection technique through in-depth interviews.

 For thematic analysis images in the results section, they cannot be changed, because the images have been registered through Figshare and uploaded directly by the F1000Research team on the paper. Furthermore, the author has also emphasized the discussion by comparing the findings and explaining which parts support and contradict the results of previous research.

 Meanwhile, in the previous conclusion (revision 2) the author has explained and answered the three research questions starting from the urgency, strategies and positive and negative impacts of the digital literacy implementation curriculum in elementary schools.

 Thank you

# Reviewer report: Reviewer response for version 2

**DOI:** 10.5256/f1000research.200592.r490724

**Published:** 2026-06-09

**Authors:** Burmansah Burmansah

**Affiliations:** 1 STIAB Jinarakkhita Lampung, Lampung, Indonesia

Thank you for the opportunity to re-review this manuscript. The authors have demonstrated a commendable responsiveness to the reviewers' comments. The structural and textual revisions have greatly improved the articulation of the arguments and the presentation of the findings. I have no further critiques and believe the paper is now well-prepared for indexing.

Is the work clearly and accurately presented and does it cite the current literature?

Partly

If applicable, is the statistical analysis and its interpretation appropriate?

Partly

Are all the source data underlying the results available to ensure full reproducibility?

Partly

Is the study design appropriate and is the work technically sound?

Partly

Are the conclusions drawn adequately supported by the results?

Partly

Are sufficient details of methods and analysis provided to allow replication by others?

Partly

Reviewer Expertise:

I am a researcher and lecturer specializing in educational sciences, learning, and teaching, particularly in the fields of educational management and leadership. My work focuses on advancing effective learning and teaching practices within educational institutions. In addition, I am a practitioner of mindfulness in education, with research interests in mindful leadership, and educational management in Buddhist institutions.

I confirm that I have read this submission and believe that I have an appropriate level of expertise to confirm that it is of an acceptable scientific standard.

# Reviewer report: Reviewer response for version 1

**DOI:** 10.5256/f1000research.189989.r438325

**Published:** 2026-01-21

**Authors:** Siti Nabilah

**Affiliations:** 1 Universitas Nahdlatul Ulama Indonesia, Jakarta, Indonesia

This study aims to examine elementary school teachers’ perspectives on the integration of digital literacy into the curriculum as a means of improving school quality.

 A quasi-qualitative approach was employed, utilizing structured interviews with 48 teachers from 26 elementary schools. The analysis identified three key themes: the level of urgency, implementation strategies, and the positive and negative impacts of digital literacy adoption. The findings indicate that digital literacy plays a crucial role in enhancing the efficient use of resources, improving educational quality, and fostering 21st-century skills.

 Therefore, the study highlights the importance of the involvement of school principals and government authorities in curriculum management, infrastructure provision, and the development of supportive policies to ensure that digital literacy implementation is sustainable and aligned with the specific characteristics of each school.

Is the work clearly and accurately presented and does it cite the current literature?

Yes

If applicable, is the statistical analysis and its interpretation appropriate?

Yes

Are all the source data underlying the results available to ensure full reproducibility?

Yes

Is the study design appropriate and is the work technically sound?

Yes

Are the conclusions drawn adequately supported by the results?

Yes

Are sufficient details of methods and analysis provided to allow replication by others?

Yes

Reviewer Expertise:

education

I confirm that I have read this submission and believe that I have an appropriate level of expertise to confirm that it is of an acceptable scientific standard.

# Response

**Published:** 2026-02-03

**Authors:** Rusi Rusmiati Aliyyah

**Affiliations:** PGSD, Universitas Djuanda, Bogor, West Java, Indonesia

Dear:

 Siti Nabilah

 F1000 Research Reviwer

 Thank you for the review and input provided, hopefully it will be an improvement for the development of my competencies.

# Reviewer report: Reviewer response for version 1

**DOI:** 10.5256/f1000research.189989.r438326

**Published:** 2026-01-09

**Authors:** Sri Marmoah

**Affiliations:** 1 Universitas Sebelas Maret, Surakarta, Central Java, Indonesia

In the **abstract**, it is important to clarify the purpose of the study. In addition, the results presented are still too basic and have not yet been able to summarize the overall findings discussed in the paper.

 In the **introduction**, it would be better to provide a positive statement related to the topic being investigated, followed by the research gap or the main issues that create the need for further study. This section should also be supported by a clear explanation of the urgency of the research. Since many similar studies have been conducted, it is necessary to explain the novelty of this study and provide relevant literature reviews.

 Furthermore, I do not clearly understand whether the discussion of the *Merdeka Curriculum* is intended as part of the literature review or as the closing section of the introduction. If it is considered part of the literature review, then the absence of a discussion on digital literacy—which is the main focus of the study—needs to be addressed.

 In the **methodology** section, the participants’ demographic details need to be explained more thoroughly, including how the 48 teachers were selected from 26 schools. The authors also need to provide justification for how the interview questions were formulated. A detailed explanation of how the in-depth interviews were conducted should be included, such as the duration of the interviews and the medium used. In addition, clarification is needed as to why only five teachers were selected, even though participants came from seven provinces in Indonesia.

 In the **results** section, the concrete manifestations of digital literacy in elementary schools are not yet clearly visible.

 In the **discussion** section, the results need to be analyzed and compared with findings from previous studies to determine whether they support or contradict earlier research.

 In the **conclusion**, the section does not yet cover all of the findings discussed. It should be able to answer the research questions or objectives and clearly state the implications of the research that has been conducted.

Is the work clearly and accurately presented and does it cite the current literature?

Yes

If applicable, is the statistical analysis and its interpretation appropriate?

Not applicable

Are all the source data underlying the results available to ensure full reproducibility?

Partly

Is the study design appropriate and is the work technically sound?

Partly

Are the conclusions drawn adequately supported by the results?

Partly

Are sufficient details of methods and analysis provided to allow replication by others?

No

Reviewer Expertise:

Educational management, elementary school

I confirm that I have read this submission and believe that I have an appropriate level of expertise to confirm that it is of an acceptable scientific standard, however I have significant reservations, as outlined above.

# Response

**Published:** 2026-04-30

**Authors:** Rusi Rusmiati Aliyyah

**Affiliations:** PGSD, Universitas Djuanda, Bogor, West Java, Indonesia

Dear:

**Sri Marmoah**

**Reviewer F1000Research**

 Thank you for the suggestions and inputs that have been given.

 Attached are revisions of articles that have been adjusted based on the suggestions given:

1. Abstracts have been added to the research objectives and explanations of the results of the research results and recommendations
2. An introduction has been given a conceptual explanation, then the problems and gaps that occur are explained, so it is important to conduct research. In this section, the urgency and novelty of research have also been given
3. In the methodology section, the reasons for determining 48 respondents from 26 schools and the selection of provinces as the place of research have been added. In this section, an explanation has also been given of how the interview was conducted in detail and an explanation of taking 5 sample teachers (respondents) for in-depth interviews.
4. The discussion of the study has been refined by providing a more in-depth explanation of the analysis of the findings
5. The conclusion has been given an in-depth explanation of the findings according to the research objectives and provides implications, suggestions and recommendations at the end

# Reviewer report: Reviewer response for version 1

**DOI:** 10.5256/f1000research.189989.r438333

**Published:** 2026-01-02

**Authors:** Burmansah Burmansah

**Affiliations:** 1 STIAB Jinarakkhita Lampung, Lampung, Indonesia

The article provides valuable insights into teachers' perspectives on implementing digital literacy in Indonesian elementary schools. Its strengths include the relevance of the topic, thematic depth, and qualitative richness through direct quotes. However, it could benefit from clearer definitions and context regarding digital literacy. The discussion needs stronger integration of existing literature and a more transparent acknowledgment of the study's limitations. Additionally, the conclusion should offer specific, actionable recommendations for schools and policymakers, as well as suggestions for future research. Addressing these areas would enhance the article's rigor and contribution to the field of education significantly.

 Introduction Section:

 1.  To enhance the thematic depth and overall quality of the article, the authors should clarify the objectives of each theme in the introduction, articulating their significance in improving digital literacy in education. Expanding the thematic analysis with comprehensive insights and direct quotes from participants will create richer narratives. Incorporating relevant literature that contextualizes each theme can strengthen the argument and connect the study to existing research. Highlighting interconnections between themes can provide a nuanced understanding, while acknowledging counterarguments will present a balanced view. Practical recommendations for schools and policymakers based on findings will enhance applicability, and concluding with the implications of these themes will reinforce the study's relevance.

 2. Enhance the introduction with clearer definitions and context about digital literacy. The introduction lacks a comprehensive definition of digital literacy. Authors should provide a clearer explanation of its key components and significance within the educational context. This enhancement will ensure readers understand its relevance to both teachers and students.

 3. The study highlights the pressing need for digital literacy in education to prepare students for the demands of the 21st century. It emphasizes that enhancing digital skills is essential for improving the overall quality of education. Discuss its key components and relevance in education. Use clear, accessible language that establishes why digital literacy is crucial for elementary education.

 Methodology Section:

 1. The quasi-qualitative design seems appropriate for understanding teachers' perceptions. However, there could be more rigor in how participants were selected. Expand the rationale for the purposive sampling method and discuss its limitations alongside its advantages. Consider specifying why these provinces were chosen.

 2. The methodology section gives a good overview, but the specifics of thematic analysis need more elaboration. Include detailed steps of how thematic analysis was conducted, such as coding procedures and how themes were derived. An example of codes and themes would enhance clarity and allow replication.

 Results Section:

 1. Look for areas summarizing the three main themes without sufficient clarity or detail. Revise to clearly label and define each theme and subtheme. Provide concise descriptions and direct quotes from participants that illustrate these themes effectively.

 2. Ensure that tables and figures, such as "Figure 1," are referenced appropriately and clearly explained. Revise to include a brief description of what the figure shows and how it relates to the themes discussed. Ensure all visual data is easily interpretable.

 3. Look for sections that present demographic data (e.g., Table 1) but lack explanation of its relevance. Include a discussion on how the demographic characteristics of participants may influence their perspectives on digital literacy, enhancing the context of the findings.

 Discussion Section:

 1. Instances where findings are presented without sufficient connection to existing literature. Revise by explicitly referencing previous studies that support or contrast with the findings. This will contextualize the results within the broader educational landscape.

 2. Look for sections discussing themes in isolation without exploring their interrelatedness. Revise to discuss how the urgency, strategies, and impacts of digital literacy are interconnected. Use transitions between themes to illustrate their relationships effectively.

 3. Discussions that do not adequately address the limitations of the study. Include a dedicated subsection that acknowledges the limitations (such as sample size or geographic focus). Acknowledging these will enhance credibility and provide transparency.

 4. The discussion may lack a section on implications for future research. Conclude the discussion with specific suggestions for future studies, such as exploring digital literacy implementation in junior high schools or assessing long-term impacts on student outcomes.

 Conclusion Section:

 The conclusion effectively summarizes the importance of digital literacy for enhancing education quality and fostering 21st-century skills. However, it could strengthen its impact by explicitly connecting the main themes: urgency, strategies, and impacts to the findings. Offering specific recommendations for schools and policymakers would enhance applicability. Additionally, acknowledging limitations discussed earlier would improve transparency and understanding of the research context. Including suggestions for future research avenues, such as examining long-term impacts on student outcomes, would provide further depth. An impactful closing statement emphasizing the transformative potential of digital literacy in education could leave a lasting impression on readers.

Is the work clearly and accurately presented and does it cite the current literature?

Partly

If applicable, is the statistical analysis and its interpretation appropriate?

Partly

Are all the source data underlying the results available to ensure full reproducibility?

Partly

Is the study design appropriate and is the work technically sound?

Partly

Are the conclusions drawn adequately supported by the results?

Partly

Are sufficient details of methods and analysis provided to allow replication by others?

Partly

Reviewer Expertise:

I am a researcher and lecturer specializing in educational sciences, learning, and teaching, particularly in the fields of educational management and leadership. My work focuses on advancing effective learning and teaching practices within educational institutions. In addition, I am a practitioner of mindfulness in education, with research interests in mindful leadership, and educational management in Buddhist institutions.

I confirm that I have read this submission and believe that I have an appropriate level of expertise to confirm that it is of an acceptable scientific standard, however I have significant reservations, as outlined above.

# Response

**Published:** 2026-04-30

**Authors:** Rusi Rusmiati Aliyyah

**Affiliations:** PGSD, Universitas Djuanda, Bogor, West Java, Indonesia

Dear:

**Burmansah Burmansah**

**Reviewer F1000Research**

 Thank you for the suggestions and inputs that have been given.

 Attached are revisions of articles that have been adjusted based on the suggestions given:

1. The introduction has been enhanced by adding definitions and context about digital literacy. In this section, an explanation of the urgency and novelty of the research has also been given
2. In the methodology section, an explanation has been given about the selection of respondents and the province that is used as the location of the research
3. In the discussion section, a more in-depth explanation has been given about the analysis of research findings
4. The researcher has refined the explanation in the section of the limitations of the research
5. The conclusion has been given a more detailed explanation of the results of the research findings and given implications, suggestions and recommendations at the end of the section

# Reviewer report: Reviewer response for version 1

**DOI:** 10.5256/f1000research.189989.r438334

**Published:** 2025-12-31

**Authors:** Esmi Tsalsa Sofiawati

**Affiliations:** 1 STKIP Bina Mutiara Sukabumi, Sukabum, Indonesia

The article is clearly written and well structured. The study design, methodology, and analysis are appropriate and sufficiently detailed to ensure replicability. Statistical analysis is correctly applied and interpreted. The conclusions are well supported by the results, and the study makes a relevant and meaningful contribution to the field and existing literature.

Is the work clearly and accurately presented and does it cite the current literature?

Yes

If applicable, is the statistical analysis and its interpretation appropriate?

Yes

Are all the source data underlying the results available to ensure full reproducibility?

Yes

Is the study design appropriate and is the work technically sound?

Yes

Are the conclusions drawn adequately supported by the results?

Yes

Are sufficient details of methods and analysis provided to allow replication by others?

Yes

Reviewer Expertise:

Education, elementary education, digital literacy

I confirm that I have read this submission and believe that I have an appropriate level of expertise to confirm that it is of an acceptable scientific standard.

# Response

**Published:** 2026-02-03

**Authors:** Rusi Rusmiati Aliyyah

**Affiliations:** PGSD, Universitas Djuanda, Bogor, West Java, Indonesia

Dear:

 Esmi Tsalsa Sofiawati

 F1000 Research Reviwer

 Thank you for the review and input provided, hopefully it will be an improvement for the development of my competencies.
